# Retinoic Acid and Its Role in Modulating Intestinal Innate Immunity

**DOI:** 10.3390/nu9010068

**Published:** 2017-01-13

**Authors:** Paulo Czarnewski, Srustidhar Das, Sara M. Parigi, Eduardo J. Villablanca

**Affiliations:** Immunology and Allergy Unit, Department of Medicine, Solna, Karolinska Institutet and University Hospital, Stockholm 171-76, Sweden; paulo.czarnewski@ki.se (P.C.); srustidhar.das@ki.se (S.D.); martina.parigi@ki.se (S.M.P.)

**Keywords:** vitamin A, retinoic acid, innate immunity, dendritic cells, innate lymphoid cells

## Abstract

Vitamin A (VA) is amongst the most well characterized food-derived nutrients with diverse immune modulatory roles. Deficiency in dietary VA has not only been associated with immune dysfunctions in the gut, but also with several systemic immune disorders. In particular, VA metabolite *all-trans* retinoic acid (*at*RA) has been shown to be crucial in inducing gut tropism in lymphocytes and modulating T helper differentiation. In addition to the widely recognized role in adaptive immunity, increasing evidence identifies *at*RA as an important modulator of innate immune cells, such as tolerogenic dendritic cells (DCs) and innate lymphoid cells (ILCs). Here, we focus on the role of retinoic acid in differentiation, trafficking and the functions of innate immune cells in health and inflammation associated disorders. Lastly, we discuss the potential involvement of *at*RA during the plausible crosstalk between DCs and ILCs.

## 1. Introduction

Vitamin A (VA) is a lipophilic micronutrient obtained by dietary ingestion of primarily pro-vitamin A carotenoids (such as β-carotene) and retinyl esters (RE) derived from plant and animal food, respectively. Humans are not able to synthesize VA and thus depend on dietary replenishment for maintenance of several physiological processes throughout the body (reviewed in [1]). After intake, carotenes are enzymatically processed in the intestine to form retinol, which is transported from the lumen into the cell cytoplasm and is rapidly converted to RE or to retinoic acid (RA) [2]. *All*-*trans*-RA (*at*RA) is the physiologically most abundant and well-studied compared to other RA isoforms such as 9-*cis*-RA and 13-*cis*-RA. Since the current review focuses on the effect of RA on intestinal innate immunity, more detailed information on VA metabolism, absorption, and transportation to the peripheral tissues have been extensively reviewed elsewhere [3,4,5,6], which is beyond the scope of this review. 

Micronutrient deficiency, in addition to protein-energy malnutrition (PEM), is being recognized as an important factor contributing towards the global burden of infectious diseases, particularly among children and pregnant women [7,8]. VA, among other micronutrients, has been closely associated with infection related morbidity and mortality in children [7]. Given the recent findings on the role of VA affecting both the innate and adaptive arms of the immune system, it seems plausible to implicate VA deficiency in increased susceptibility towards infectious diseases [7,8]. On the other hand, VA excess leads to deregulation of liver metabolic functions and presents several other toxic effects [9]. The role of VA metabolite *at*RA in adaptive immune responses has been extensively reviewed elsewhere [10,11,12], particularly in the context of intestinal immunity. Here, we review the recent findings where *at*RA plays a central role in the development and functioning of the innate arm of the immune system, in particular the myeloid compartment and innate lymphoid cells (ILCs). 

## 2. Sources of *at*RA

A major fraction of the dietary VA is stored in the hepatic stellate cells with the ability to transform VA/retinol to *at*RA [13,14]. However, in the intestine where dietary VA is absorbed and assimilated, several cell types are capable of metabolizing retinol to *at*RA. The conversion of retinol to retinaldehyde is mediated by alcohol dehydrogenases (ADHs), which are ubiquitously expressed, whereas the subsequent oxidation of retinaldehyde to *at*RA is mediated by one of the three retinaldehyde dehydrogenases (ALDHs) isoforms (ALDH1A1, ALDH1A2 and ALDH1A3), the expression of which is more restricted to *at*RA-producer cells [15]. In addition, RA can be degraded through cytochrome P450 family 26 (CYP26 family), the expression of which is distributed over several tissues and cell types [16,17]. Although *at*RA can influence the immune system in multiple organs, the intestinal immune system is one of the most affected and well-characterized ones [18]. Therefore, we have primarily focused on sources of *at*RA that affect the intestinal immune system. 

Besides being the primary site for VA absorption, intestinal epithelial cells (IECs) can metabolize VA to RA by their ability to express ALDH1A1 [19,20]. However, our understanding of which IEC types are primarily responsible for metabolizing VA to RA is not complete. In addition, a subset of lamina propria stromal cells (LP-SCs) underlying IECs also express all ALDH isoforms and are able to metabolize VA [21]. Our knowledge about *at*RA-producer cells flourished after ALDEFLUOR (ALDE), a commonly used reagent in stem cell research, was used to label cells containing ALDH activity [22]. Using ALDE combined with specific cell surface markers, it is possible to determine that ALDH activity is restricted specifically to CD103^+^ DCs but not CD103^−^ DCs or any other hematopoietic-derived immune cell types in the intestine. This is in agreement with the expression of *Aldh1a2* transcripts found uniquely on CD103^+^ DCs. Interestingly, DCs located at the proximal small intestine exhibit higher ALDH activity compared to DC located in the distal small intestine or colon [15,23], likely due to increased absorption of VA in the proximal part of the intestine. This correlates with gut-associated lymphoid tissue (GALT) DC functions, as seen in experiments in which DCs isolated form the proximal small intestine induced higher levels of Foxp3^+^ T_REG_, gut homing molecules such as the CCR9 chemokine receptor and α4β7 integrin on T cells, and IgA class switching on B cells compared to DCs isolated from distal intestinal tissues [15]. Interestingly, CD103^+^ DCs are characterized by their ability to migrate towards the mesenteric lymph nodes (MLN) where these DCs are one of the predominant sources of *at*RA, important for generating gut-tropic T cells in mice [24,25]. 

Within MLN, multiple sources of *at*RA have been proposed, of which stromal cells and CD103^+^ DCs are the best characterized [24,25,26]. The important contribution of *at*RA produced by MLN-derived stromal cells (MLN-SCs) stems from the study by the Oliver Pabst group in which peripheral lymph nodes (PLN) that do not express *at*RA-synthesizing enzymes when transplanted into the mesenteries resulted in diminished induction of gut-homing T cells [26]. In these experiments, even if the transplanted PLN is reconstituted with gut-derived DCs, the induction of gut-tropism was abolished [26], suggesting that *at*RA and/or other metabolite(s) produced specifically by resident MLN-SCs are critical for the induction of gut-homing receptors in T cells. In agreement, only MLN-SCs express *at*RA-synthesizing enzymes and support gut homing induction on T cells in vitro [26]. Interestingly, in the same study, bone marrow derived DCs (BM-DCs) that do not express *at*RA-synthesizing enzymes failed to induce gut homing receptors when injected directly into the MLN of CCR7-deficient mice [26]. This indicates that MLN-SC derived *at*RA is not sufficient for inducing gut tropism and may depend on lamina propria-derived DCs (which are absent in the C-C chemokine receptor type 7 (CCR7)-KO mice). Similarly, T cells adoptively transferred into CCR7-KO mice did not express gut-homing markers upon activation [27]. Further support towards the role of MLN-DCs stems from studies where MLN-DCs, characterized by the expression of *at*RA-synthesizing enzymes, are sufficient to induce gut homing receptors on T cells in vitro [28,29]. Are *at*RA-producer DCs sufficient for inducing gut tropism in vivo? Our unpublished data showed that subcutaneous injection of antigen-bearing *at*RA-producer DCs (e.g., MLN-DCs) were able to induce antigen-specific T cell activation and proliferation within the PLN but failed to induce gut-homing receptors, suggesting that DCs depends on the environment to induce gut tropism in vivo. This observation favors the notion that stromal cells from the MLN are essential to support *at*RA-mediated gut homing induction [26]. Another interpretation of these results is the possibility that the PLN displayed microenvironment-derived inhibitory signals towards the induction of gut homing receptors. Indeed, prostaglandin E_2_ (PGE_2_) has been shown to inhibit the induction of *at*RA-synthesizing enzymes (e.g., *Aldh1a2*) in DCs, and reduction of PGE_2_ synthesis in vivo results in the emergence of systemic *at*RA-synthesizing DCs [30]. It would be interesting then to explore the possibility of using gut-associated DCs as intradermal vaccines in the presence of PGE_2_ inhibitor to induce gut immune responses. Thus, it is likely that both MLN-SCs and DCs combine their functions to effectively produce and provide *at*RA and the consequent induction of gut-homing receptors on lymphocytes. 

In summary, although VA can be metabolized to *at*RA by IECs, LP and MLN DCs, LP and MLN stromal cells, whether the contribution from each of these known players is sequential, cooperative and/or mutually exclusive needs further investigation. We hypothesize that whether *at*RA produced by one cell type is sufficient or needs to act together with other cell types depends largely on the biological context (e.g., steady-state versus disease). For instance, atRA produced by IECs may be sufficient for influencing colorectal cancer [19], whereas it might be required in both DCs and stromal cells to affect T cell priming and gut-homing [26,28].

## 3. RA and Intestinal Homeostasis

Among many nutrients and metabolites digested on a daily basis, VA orchestrates intestinal homeostasis and, in particular, intestinal immune homeostasis at multiple nodes [31]. Both the non-immune and immune cells in the gut are capable of producing and/or sensing *at*RA and therefore can contribute to the overall intestinal homeostasis. While the production of RA by multiple intestinal cell types depends on their ability to express ALDHs, the ability to sense RA is dependent on the expression of receptors that are broadly classified into two sub groups, i.e., retinoic acid receptor (RAR) and retinoid X receptor (RXR). Furthermore, each sub group is comprised of three sub types such as RAR α/β/γ and RXR α/β/γ [2,32]. Both *at*RA and 9-*cis*-RA can bind to and activate RAR that forms a heterodimer with the RXR, which then leads to transcriptional regulation of gene expression by binding to retinoic acid response elements (RARE). RXR, on the other hand, can be activated only by 9-*cis*-RA, although it can partner with several other nuclear receptors such as liver X receptor (LXR), peroxisome proliferator-activated receptor gamma (PPARγ) etc. and affect gene expression [32]. The ability of various cell types in the intestine to sense and respond to RA is described in the following section. 

Besides their role as a physical barrier, IECs can sense the luminal content and play essential roles in imprinting the underlying innate immune system [2], although intestinal epithelium is the first line the dietary metabolites encounter, if *at*RA acts on intestinal epithelial cells to modulate their development or function is poorly understood. Interestingly, experiments using zebrafish larvae suggest that *at*RA inhibits the differentiation of intestinal epithelial cells with mucosecretory phenotype, presumably goblet cells [33]. In agreement, unpublished data from our laboratory show that mice lacking RARα, specifically in IECs, result in goblet cell numbers that significantly outnumber their WT counterparts, suggesting a direct role of *at*RA in the intestinal epithelial cells’ differentiation program. Although the effect of *at*RA in IECs is still an unexplored area, the ability of IECs to metabolize *at*RA has been extensively documented [1,2,3,19]. The recent work from Engleman and colleagues clearly demonstrated that healthy human colonic epithelium produce *at*RA, in contrast to the epithelium from inflammation-driven colorectal cancer patients that were compromised in their ability to produce *at*RA. This was shown to be a result of dramatic decrease in the expression of *at*RA synthesizing enzymes ALDH1A1 with concomitant increase in *at*RA degrading enzyme CYP26A1 in intestinal tissues from tumor-bearing compared to tumor-free mice [19]. Importantly, *at*RA supplementation reduced the tumor burden in a mouse model of colorectal cancer [19], highlighting the relevance of VA as a potential therapeutic option to treat colon cancer patients. In addition to the effects of *at*RA directly on the IECs, the epithelium mediates many of the immune responses by acting as a sensor of the environment and delivering cytokines and/or factors that modulate immune cell functions underlying the epithelial layer [34]. For instance, IEC produced *at*RA have been suggested to imprint tolerogenic functions in dendritic cells, a key step in the induction of oral tolerance to luminal antigens [15,20,35].

A major advance in our understanding of VA as an immune modulator occurred in 2004 when Iwata and colleagues demonstrated that *at*RA was necessary and sufficient to induce the gut homing receptors, CCR9 and α4β7 on T cells [28]. This work was followed by Mora et al., showing that *at*RA induces gut tropism also on B cells [36]. Importantly, mice fed with VA-depleted (VAD) diet are almost devoid of CD4^+^ and CD8^+^ T as well as B cells in the small intestine lamina propria [28,36], highlighting the physiological importance of VA in intestinal lymphocyte homeostasis. Furthermore, intraepithelial lymphocytes (IELs), which are found abundantly in proximity to the intestinal epithelial cells, also rely on gut homing receptors to infiltrate the small intestine and position themselves at the epithelial site [37]. In agreement, mice expressing a dominant-negative form of the RARα, specifically in CD4^+^ T cells, lack the CD4^+^ IEL population [38]. VA has also been shown to affect IgA secreting intestinal B cell homeostasis. For example, VA-deficient mice lack IgA antibody-secreting cells (IgA-ASCs) in the intestinal lamina propria. This has been attributed to a combinatorial effect of lack of *at*RA on impaired induction of gut homing receptors on IgA secreting B-cells and inability to synergize with the IL-6 or IL-5 production by DCs and/or other cells types [36]. In agreement, experiments depleting RARα specifically in B cells render mice with intestinal microbiota dysbiosis and failure in developing adequate immune responses after oral immunization [39]. Thus, *at*RA has been shown to play important roles regulating adaptive immunity. Since the role of *at*RA modulating T cell homeostasis has been extensively reviewed elsewhere [10,11,12], we will further discuss its role as a modulator of the innate immune arm.

## 4. RA in Dendritic Cells (DCs)

Dendritic cells are characterized by their ability to sense and process antigens at peripheral tissues, and they migrate towards draining lymph nodes, produce specific cytokines and prime the differentiation of antigen-specific lymphocytes [40]. Hence, dendritic cells are specialized sentinels of our immune system capable of orchestrating the innate and adaptive immune response. Several of these functions of DCs are tightly regulated by *at*RA that acts at multiple nodes by influencing their differentiation and function. Although the majority of the studies are focused on intestinal DCs, some reports highlighted the role of *at*RA on myeloid cells beyond the intestine [41,42]. In the following section, we will discuss the effect of *at*RA in influencing DC differentiation from the bone marrow and their function in the periphery.

**Figure 1:**
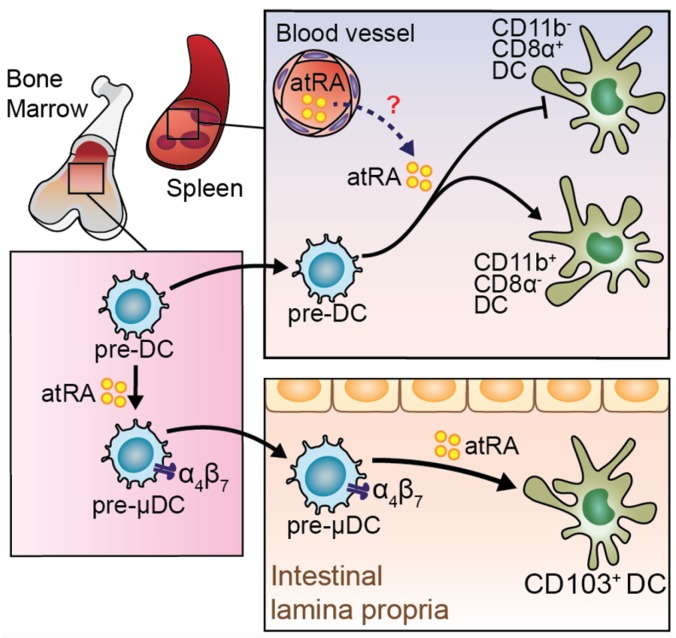
Role of *all-trans* retinoic acid (*at*RA) in regulating differentiation of dendritic cells (DC) precursors. Bone marrow (BM) resident pre-DCs have the potential to differentiate into pre-mucosal DC (pre-μDC), characterized by the expression of gut homing receptors. Expansion of pre-μDC is *at*RA-dependent. Pre-μDC gives rise to intestinal CD103^+^DCs, which is enhanced by the presence of *at*RA. Pre-DCs can migrate to the spleen, where they may sense *at*RA skewing the differentiation toward CD11b^+^CD8^−^ DCs instead of CD11b^−^CD8α^+^ DCs.

Based on their expression of α4β7 and their ability to preferentially give rise to intestinal CD103^+^ DCs and CCR9^+^ plasmacytoid DCs, a specialized subset of precursors named pre-µDCs (pre-mucosal DCs) has been identified in murine lymphoid organs and in the bone marrow [43] (Figure 1). In vitro differentiation studies proved the ability of *at*RA to dramatically expand pre-µDCs among the total BM progenitors. In line with these findings, mice fed with a VAD diet resulted in a significant decrease in the BM pre-µDCs pool [43]. This phenotype was coupled in the periphery by a selective reduction of cDC2 (or CD103^+^CD11b^+^ DCs) in the intestine and GALT [44] (Figure 1) and of the developmentally related CD11b^+^ CD8a^−^Esam^hi^ DC population in the spleen [45]. 

One of the current challenges in translational mucosal immunology is to identify the most efficient protocol to generate gut-like DCs that could be used as peripherally administered vaccines, enabling us to generate efficient mucosal immune responses. In vitro differentiation of pre-µDCs in the presence of Fms-related tyrosine kinase 3 ligand (Flt3L), and granulocyte-macrophage colony-stimulating factor (GM-CSF) was not sufficient to recreate bona fide intestinal-like DCs [44]. Remarkably, addition of *at*RA was shown to direct the generation of DCs that mimicked the transcriptomic profile particularly of in vivo intestinal cDC subsets [44]. However, whether such in vitro generated DCs are functionally as competent as the in vivo cDCs remains to be tested. 

After differentiation and migration to the intestine, specific DC subsets are endowed with the ability to sense and respond to *at*RA [45]. The general consensus on the effect of *at*RA on DC function is to promote an anti-inflammatory phenotype characteristic of intestinal DCs [46,47]. Transcriptional profiling of in vitro differentiated DCs in the presence of *at*RA showed downregulation of pro-inflammatory genes involved in the NF-kB mediated inflammatory program [45]. However, besides the above-mentioned immuno-regulatory effects, some studies have emphasized a rather unorthodox pro-inflammatory role of *at*RA in DCs. In the presence of IL-15, *at*RA was shown to act as an adjuvant in promoting the secretion of the pro-inflammatory cytokines IL-12 and IL-23 by DCs [48]. Similarly, human DCs generated from monocytes in the presence of *at*RA primarily induced interferon gamma (IFNγ) production by CD4^+^ T cells in vitro [49]. The dual role of *at*RA in influencing DCs function might be a result of the microenvironments and/or cytokine milieu to which the DCs are exposed. For example, *at*RA in a cytokine environment that is either pro- or anti-inflammatory would induce a tolerogenic or pro-inflammatory DC phenotype, respectively. 

Besides being able to sense and get influenced by *at*RA, DCs, in turn, are one of the main producers of *at*RA, a phenomenon that has been well-studied in intestinal CD103^+^ DCs expressing high levels of the *Aldh1a2* gene. This is most evident in the intestine where CD103^+^ DCs in the proximal tract of the small intestine are exposed to higher levels of *at*RA, which, in turn, renders them better *at*RA producers compared to distal small intestinal or colonic DCs. However, this is not limited to the intestine since extra intestinal DCs endowed with the ability to produce *at*RA have also been described, such as CD103^−^ DCs in the skin [41]. Importantly, CD103^−^ DCs in the skin can induce Foxp3^+^ T_REG_ similar to intestinal CD103^+^ DCs [41]. Several factors can influence DCs into *at*RA-producing cells. For example, incubation of DCs with *at*RA alone or in combination with various cytokines such as interleukin-4 (IL-4) and transforming growth factor beta (TGFβ) cytokines were able to induce *Aldh1a2* mRNA expression, which seems to be sufficient to render DCs with the capacity to metabolize *at*RA [50,51,52]. In addition, the short chain fatty acid butyrate was shown to induce *Aldh1a* expression in monocyte-derived dendritic cells, suggesting that microbiota-derived metabolites might play important roles in intestinal DC function [53]. Moreover, the Wnt pathway seems to be crucial in imprinting intestinal DCs since this pathway seems to be crucial to induce *Aldh1a2* expression and produce *at*RA [54]. Furthermore, MyD88 signaling and, in particular, toll-like receptor (TLR) 1/2 stimulation can also induce *Aldh1a2* mRNA expression and imprint extra-intestinal DCs with *at*RA producing capacity [15,55,56]. While multiple factors and pathways have been shown to influence the *at*RA producing capacity of DCs, whether two or more of these pathways interact with each other needs to be investigated. Irrespective of the mechanism, DCs imprinted to produce *at*RA primarily induce a tolerogenic program by generation of Foxp3 T_REG_ cells. Importantly, *at*RA production by intestinal CD103^+^ DCs seems to be crucial for the generation of T_REG_ and for the establishment of tolerance towards innocuous dietary antigens, a process known as oral tolerance.

## 5. *at*RA-Producer Dendritic Cells in Oral Tolerance

**Figure 2:**
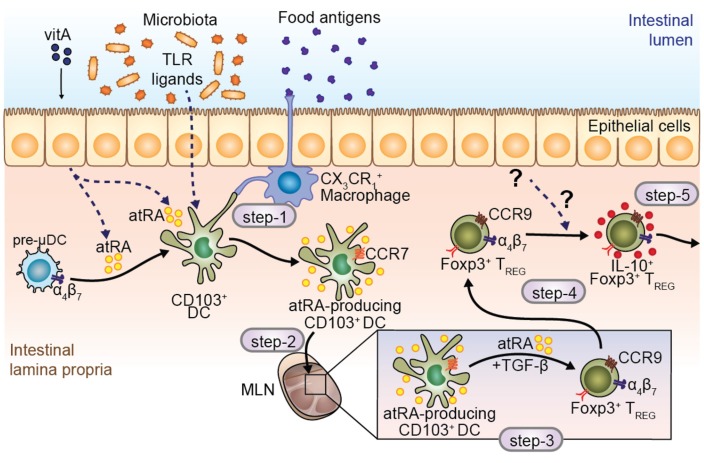
Role of *at*RA in modulating intestinal DC differentiation, maturation and function. CD103^+^ DCs reside beneath the epithelial layer, in close contact with CX3CR1^+^ macrophages, which are responsible for taking up luminal antigens and transferring them to CD103^+^ DCs (step-1). Several molecules are described to induce *at*RA-producing CCR7^+^ DCs, such as toll-like receptor (TLR) ligands and *at*RA itself, making DCs equipped to migrate to the mesenteric lymph nodes (MLN) (step-2). Within the MLN, CD103^+^ DCs are responsible for antigen presentation and differentiation of regulatory T cells in the presence of *at*RA and TGF-β (step-3). Moreover, DC-derived *at*RA is crucial to induce the expression of gut homing receptors in T cells (step-3). Finally, regulatory T cells reach the intestinal mucosa and gain the capacity to produce IL-10, becoming IL-10 producing T_REG_, which play a key role in the establishment of oral tolerance in the intestinal lamina propria (step-4); T_REG_ induced in this manner are able to migrate to peripheral tissues and promote tolerance (step-5). Steps 1 to 5 denote the main processes involved in the establishment of oral immunological tolerance.

Local and systemic immune unresponsiveness towards antigens that have been previously administered by oral route is classically defined as oral tolerance [12,57]. These antigens, which are considered innocuous, are primarily derived from diet and failure to mount an effective tolerance towards these antigens has been associated with conditions such as food allergies and celiac disease. VA has proven crucial to the establishment of oral immunological tolerance against food antigens [12,57], and its deficiency might contribute towards food allergies, celiac disease and inflammatory bowel diseases (IBD). Indeed, mice deprived of VA resulted in exacerbated intestinal inflammation as assessed by colitis score and colon length using the dextran sodium sulfate (DSS)-induced colitis model [58]. Moreover, *at*RA supplementation efficiently attenuates chronic inflammation in a mouse model of ileitis [59]. Notably, *at*RA participates in more than one of the five-step models proposed for the establishment of oral immunological tolerance [12] (Figure 2).

**Figure 3:**
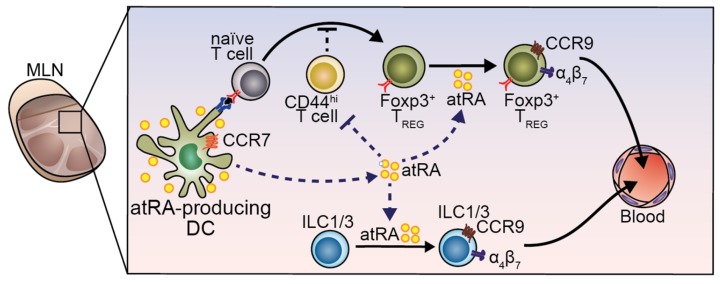
Role of DC-derived *at*RA in induction of gut-homing T_REG_ and innate lymphoid cells (ILCs). Mature DCs arrive in the MLN and are capable to produce *at*RA, as well as other stromal cells. *at*RA is important for generating T_REG_ by attenuating CD44^hi^ cells that inhibit T_REG_ differentiation. In addition, *at*RA in the MLN is crucial for induction of gut-homing receptors in T cells and in ILC1 and ILC3.

The first step involves pick up of antigens by CX_3_CR1^+^ macrophages and transfer to CD103^+^ DCs [60] or by CD103^+^ DCs themselves [61] (Figure 2; step-1), which later will migrate towards the mesenteric lymph node (MLN). To reach the MLN (Figure 2; step-2), CD103^+^ DCs need to enter into the lymphatic vessels and move from CCL21-Leu to CCL21-Ser gradient present in the lymphatic endothelium in a chemokine receptor 7 (CCR7) dependent manner [62,63]. However, if *at*RA is essential for CCR7-dependent migration of DCs in vivo, is still unknown. Once in the MLN, antigen-bearing CD103^+^ DCs encounter and prime antigen specific naïve T cells, which will differentiate to activated T helper cells. Depending on the microenvironment and cytokine milieu, T cells can become, among others, pro-inflammatory Th17 cells or immunosuppressive Foxp3^+^ regulatory T cells (T_REG_). During the establishment of oral tolerance, in step-3, the generation of antigen-specific Foxp3^+^ T_REG_ is mandatory (Figure 2; step-3), in which stimulation of the T cell receptor (TCR), IL-2R and the TGF-β receptor are required. In this setting, *at*RA acts as an adjuvant by enhancing the generation of Foxp3^+^ T_REG_ cells through the inhibition of Foxp3-inhibitory cytokine production by effector CD44^hi^ T cells [64] (Figure 3). In addition to enhanced T_REG_ differentiation, *at*RA may restrict Th17 differentiation [35,65,66] and indirectly suppress T_H_2 inflammatory responses (reviewed in [10]) that may eventually favor the balance towards the generation of Foxp3^+^ T_REG_ cells. Indeed, a recent study showed that treatment of T_REG_ cells with *at*RA was able to preserve Foxp3 expression in these cells under Th17 polarizing conditions compared to the control [67]. In agreement, supplementation of *at*RA has been found to suppress T_H_2 and T_H_17 mediated inflammation in a mouse model of airway allergy [68,69]. However, whether *at*RA directly inhibits naïve T cells into T_H_2 phenotype or whether it enhances T_H_2-supressor cells needs to be further explored. 

Interestingly, the crucial role of *at*RA in step-3 of oral tolerance establishment seems to be more related to the induction of gut-homing receptors rather than the generation of Foxp3^+^ T_REG_ per se. Once Foxp3^+^ T_REG_ expresses the integrin α4β7 and the chemokine receptor CCR9 are generated in the MLN in an *at*RA-dependent manner, they are equipped to migrate towards the small intestine lamina propria [70], where they will expand and acquire the ability to produce IL-10 [70,71] (Figure 2; step-4). Fully differentiated IL-10 producing T_REG_ can enter the blood circulation and immunosuppress effector immune responses in peripheral tissues (Figure 2; step-5). Interestingly, mice fed with a VAD diet are not able to establish tolerance to orally administered antigens. Cassani et al. showed that adoptive transfer of in vitro generated antigen-specific gut-tropic CD4^+^ T cells into VAD mice was able to rescue oral tolerance to ovalbumin. This indicates that *at*RA is required for the induction of gut-tropism rather than expansion or full differentiation of Foxp3^+^ T_REG_ at the lamina propria [70]. Moreover, CCR9-deficient Foxp3^+^ T_REG_ were not able to establish oral immunological tolerance [70], suggesting that *at*RA might be critical in inducing gut tropism rather than Foxp3^+^ T_REG_. All together, these data suggest that *at*RA plays a critical role in inducing gut tropism on T_REG_, which is an absolute requirement for establishing oral tolerance towards food antigens.

## 6. RA in Regulation of ILC Development and Function

Innate lymphoid cells (ILCs), a heterogeneous class of lymphocytes, are shown to be an important gatekeeper of immune homeostasis at the barrier sites of the body both in humans and mice [72]. The ILC family comprises NK (natural killer) cells and non-cytotoxic helper-like ILCs, distinguished by functional activity and developmental pathway. Based on cytokine production and key transcription factor expression, non-cytotoxic ILCs are further subdivided into three main subsets: ILC1 cells expressing T-bet mediate intracellular bacterial immunity by producing IFN-γ and TNF-α; ILC2 cells expressing GATA-3 are involved in immunity to helminthes, asthma and allergy through the production of IL-4, IL-5 and IL-13; and ILC3 cells expressing RORγt that can further be subdivided into LTi (lymphoid tissue inducer cell, described in a separate section below) and cells producing IL-17, IL-22 and GM-CSF contributing to intestinal homeostasis and protection towards extracellular bacteria [73]. Unlike T cells, the lineage specification decision does not require antigen-specific priming. Instead, it is decided during development from ILC-committed progenitors in the bone marrow [74]. 

**Figure 4:**
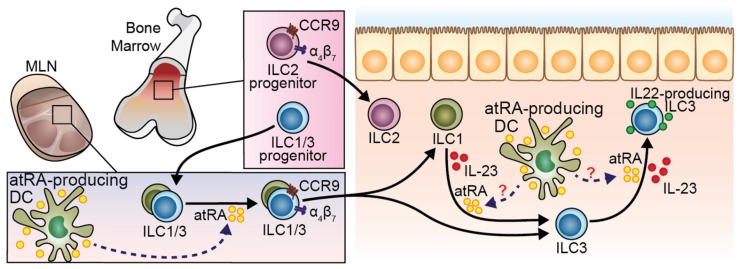
Role of *at*RA modulating ILC migration and function. ILC2 progenitors in the bone marrow (BM) express gut-homing receptors and migrate to the intestine independently of *at*RA. On the other hand, ILC1 and ILC3 progenitors deploy from the BM to the MLN. DC-derived *at*RA induce the expression of gut-homing molecules on ILC1 and ILC3 there, conferring the ability to migrate to the intestinal mucosa. Once in the gut tissue, ILC1 can potentially differentiate into ILC3 in the presence of IL-23. This plasticity may be enhanced by IL-1β and *at*RA. IL-23 induces IL-22 production by ILC3, which can be enhanced by *at*RA.

VA and *at*RA have been extensively studied as regulator of ILC development and function. Similar to T cells, ILC migration to the intestine upon development and maturation can be tightly regulated by *at*RA. Common ILC precursors (CHILPs) in the bone marrow are equipped with α4β7 integrin expression [75]. However, whether this phenomenon is *at*RA-dependent remains unaddressed. While BM-resident ILC2 committed progenitors (ILC2P) can directly migrate to the gastrointestinal tract in a CCR9- and β7-dependent way, ILC1 and ILC3 migrate first to the MLN in a CCR7-dependent manner where they undergo homing receptors switching to then migrate to the gut [76] (Figure 4). Homing tropism switch in ILC1 and ILC3 has been demonstrated in co-culture experiments, in which MLN DC-derived *at*RA was responsible for CCR7 downregulation and α4β7 and CCR9 upregulation [75]. Nevertheless, whether the interaction between ILCs and DCs takes place in vivo in the MLN remains to be unraveled. Paralleling these results in mice, in vitro experiments using human fluorescence-activated cell sorting (FACS)-purified ILCs from peripheral blood proved the ability of atRA, in concert with IL-2, to induce α4β7 [77]. Of note, this effect was abolished when the hormonally active form of vitamin D, 1,25-dihydroxyvitaminD_3_ (1,25-(OH)_2_D_3_) was added to the culture [77], suggesting a potential antagonistic effect of these two vitamins on gut-homing induction. Interestingly, VA sufficiency was proven to be dispensable for the expression of gut-homing receptors on bone marrow ILC2P [75] and rather act as a negative regulator of ILC2 differentiation [78]. ILC2P or mature ILC2 treated with *at*RA resulted in reduced expression of IL-7Ra and subsequent proliferative potential [78]. Consistently, mice fed with a VAD diet displayed increased ILC2 in the intestinal lamina propria with the consequent improved ILC2-mediated resistance to helminthic infection [78].

RA is also instrumental in fine tuning functional activity of mature ILCs in the tissue. By enhancing the production of IL-22 in natural cytotoxicity receptor (NCR)^+^ ILC3 and lymph node-derived γδ T cells, *at*RA was shown to attenuate colonic inflammation provoked by DSS administration or by *Citrobacter rodentium* infection [78,79]. The proposed mechanism was a direct binding of RARs to the Il-22 promoter, thus directly regulating Il-22 mRNA transcription [79]. However, while a putative RARγ motif was bound by RARs in the IL-22 promoter, the main isoform found to be enriched on mouse NCR^+^ ILC3 [79], and its human equivalent NKp44^+^ ILC3 [80], was RARα. Therefore, which is the relative contribution of these two isoforms in regulating IL-22 expression in ILCs still remains to be addressed. Besides regulating the functions of differentiated ILC3, *at*RA has been shown to foster the plasticity between mature ILC subsets. Indeed, in vitro studies demonstrated that *at*RA, in concert with IL-23 and IL-1β, accelerates the conversion of ILC1 to ILC3 and their IL-22 production potential [80] (Figure 4). Interestingly, this process was recreated by simply co-culturing ILC1 with intestinal CD14^−^ DCs, further supporting the hypothesis of a central role of DCs in directing ILCs function through RA [80]. 

It is well established that the concentrations of *at*RA in the small intestine follows a proximal (i.e., duodenum) to distal (i.e., colon) decreasing gradient [15,23]. Similarly, ILC subsets are differentially distributed along the gastrointestinal tract with ILC3 outnumbering ILC1 and ILC2 in the small intestine, whereas ILC2 are predominant in the colon [78]. Therefore, it is tempting to speculate that *at*RA might be one of the players involved in the complex functional regionalization of ILCs in the intestine, likely by controlling their migration, differentiation and/or function.

## 7. RA Influence on Lymphoid Organogenesis

Lymphoid organogenesis is a stepwise process that occurs during embryonic development and involves the concerted activity of different cell types [81]. RA sufficiency throughout the entire process has been shown to be instrumental and influences the function(s) of the different immune and non-immune players involved. The earliest step in lymphoid organogenesis requires the expression of the chemokine *Cxcl13* by mesenchymal organizer cells [82]. In vitro and in vivo studies demonstrated that *at*RA is pivotal in inducing *Cxcl13* expression through RARβ activation with consequent formation of the lymph node anlagen [82]. Notably, *at*RA required for this process is most likely produced by nerve fibers expressing *Aldh1a2* that colocalize with stromal cells in the anlagen [82]. Upon sensing of C-X-C motif chemokine ligand 13 (CXCL13), LTi cells are attracted towards the primordial lymphoid structure and contribute to lymphoid organogenesis by communicating with surrounding stromal cells through lympotoxin-α/β expression. LTi cells belong to the class of ILC3 and are instrumental for the formation of lymph nodes such as Peyer’s Patches (PP) in the small intestine [83], and cryptopatches and isolated lymphoid follicles in the colon [84]. Similar to other ILC3 subsets, they express the transcription factor RORγt that has been proven to be crucial for their generation. Mice deficient in RORγt are characterized by the absence of PP, PLN and MLN [83]. LTi are also characterized by the expression of RA receptors (RARα, RARβ and RARγ) and conditional deletion of RARα in fetal LTi intrinsically affects their differentiation and subsequently the development of secondary lymphoid organs [85]. Mechanistically, a direct regulation of Rorγt expression by *at*RA responsive elements upstream and within the *Rorc* locus has been suggested [85]. Remarkably, *at*RA influences LTi prenatally and the maternal dietary intake of VA or *at*RA levels in utero are fundamental to controlling the size of the lymphocyte pool and the resistance to infection in the offspring [85]. In addition to LTi cells, DCs have been recently shown to be crucial to inducing PLN maturation. It was shown that fungi-sensitized intestinal CD103^+^ALDH^+^ DCs can migrate to and promote maturation of neonatal PLN by downregulating MAdCAM-1 and upregulating PNAd expression in PLN endothelial cells, which, in turn, allow its maturation and homing of T cells to the small intestine [86]. Taken together, these studies emphasize the importance of dietary VA and RA as non-redundant early building blocks for a functional immune system.

## 8. Conclusions

Recent studies support the role of *at*RA as an important enhancer of DC differentiation and migration from the bone marrow to the intestine, ultimately resulting in DCs that are capable of both sensing and producing *at*RA. In addition, *at*RA works as a crucial regulator of DC function, which dictates T helper and effector cell function in the mucosal sites and in peripheral tissues. Interestingly, the cytokine milieu can influence DCs to induce pro-inflammatory T helper functions, even in the presence of *at*RA. Finally, recent advances in the ILC field point towards *at*RA-producing DCs as modulators of ILC migration, function and phenotype plasticity. Taken together, *at*RA exerts a crucial role in DC function in order to maintain tolerance against food and microbial antigens and promote tissue homeostasis.
